# Behaviour change interventions to promote household connectivity to sewer: a scoping review

**DOI:** 10.1080/16549716.2025.2476335

**Published:** 2025-03-21

**Authors:** Mahbub-Ul Alam, Md. Assaduzzaman Rahat, Shahpara Nawaz, Nishantika Neeher, Kazy Farhat Tabassum, Tasnia Alam Upoma, Abul Kamal, Barbara Evans, Paul Hutchings

**Affiliations:** a School of Civil Engineering, University of Leeds, Leeds, UK; b Environmental Health and WASH Research Group, Health Systems and Population Studies Division, International Center for Diarrhoeal Disease Research, Bangladesh (icddr,b), Dhaka, Bangladesh

**Keywords:** Sewer connection, sanitation, faecal waste, black water, wastewater

## Abstract

Households without access to a functioning and well-managed sanitation system produce untreated faecal waste. While connecting households to sewers is ideal in densely populated low-income areas, the connection rates often remain low. Most interventions to increase connectivity focused on addressing financial, social, and legal barriers; there is limited evidence on the effectiveness of behaviour change interventions in promoting sewer connections. Thus, we aim to understand the effectiveness of behaviour change interventions in increasing the uptake of sewer connections. We developed a review protocol with key search terms relating to households, sewers, behaviour change interventions, promotion, and effectiveness. We aimed to identify both the types of interventions deployed and their impact on increasing household sewer connections. Eleven articles met the eligibility criteria and were included in the review. Findings indicate that changes in rates of connection were associated with interventions that included a blend of indirect financial subsidy in the form of a free connection and community-engagement activities. There was limited evidence that behaviour change campaigns without financial incentives lead to changes in sewer connection rates. A multi-component package involving financial subsidies with community engagement is likely to improve the sewer connection rate.

## Paper context

- **Main findings**: Behaviour change interventions combining financial subsidies with community engagement significantly improve household sewer connection rates, while stand-alone behaviour change campaigns have limited impact.
- **Added knowledge**: This study demonstrates the critical role of multi-component interventions, integrating financial incentives and community participation, in promoting sewer connections in low-income, densely populated areas.
- **Global health impact for policy and action**: Policymakers should prioritise implementing multi-component interventions that combine financial subsidies for sewer connections with community engagement strategies, tailoring approaches to local socio-economic and cultural contexts to maximise sanitation uptake and health benefits.

## Background

Nearly half of the global population still lacks access to safe sanitation services, and the majority of those without access are poor [1]. In 2020, only 34% of the global population had safely managed sanitation through sewer connections, which were mostly prevalent in urban areas and in higher-income countries [1]. The WHO-UNICEF Joint Monitoring Programme for Water Supply, Sanitation and Hygiene (JMP) defines safely managed sanitation facilities as use of improved facilities that are not shared with other households and where excreta are safely disposed of in situ or removed and treated off-site [2]. A safely managed sanitation facility is a prerequisite to prevent exposure to excreta and ensure hygienic management and disposal of the treated excrement [3]. Households without connection to a functioning and well-managed sanitation system produce untreated faecal waste and domestic greywater (hereafter referred to as ‘wastewater’) [4]. This wastewater is typically collected in poorly constructed and improperly maintained pits and tanks, from where it is discharged either directly into storm drains or into the subsoil [5,6]. Overall, more than 80% of wastewater created by human activities is disposed into rivers and oceans without treatment, causing eutrophication, water quality deterioration, biodiversity loss, and physiological and behavioural change in existing aquatic species, which results in environmental degradation [7]. The release of untreated wastewater also contributes to the global burden of disease related to inadequate WASH, which Wolf et al. (2023) estimate is associated with 69% of diarrhoea, 14% of acute respiratory infections, and 10% of undernutrition, and approximately 100% of the burden of soil-transmitted helminthiasis [8]. There are many places where sewers exist and could convey waste to treatment, but the connection rates remain low, so the benefits of the sewers are not realised [9]. Therefore, in these areas where sewers are underutilised, interventions to encourage households to connect are practical options to improve public and environmental health [10].

Conventionally, the terms on-site and off-site sanitation systems are used widely to define excreta and wastewater management processes. In an on-site sanitation system, excreta and wastewater are collected and stored where they are produced, as opposed to off-site sanitation, which comprises a sewer network that conveys sewage to a wastewater treatment plant [11]. Sewers may be ‘separate’ or ‘combined’ – carrying wastewater exclusively in the former case or also conveying stormwater in the latter [9]. Conventional sewer design uses standard hydraulic assumptions and safety factors which result in relatively large sewers and deep excavation. Costs can be reduced through the use of ‘simplified hydraulic designs’ (simplified sewers, sometimes known as condominial sewers) or the inclusion of a settling tank at the household level (‘settled’ sewers). In both these latter cases, the resultant networks have reduced depth, smaller diameters, and shallower hydraulic gradients [9]. However, where connecting households to off-site sanitation (sewer) is regarded as an appropriate waste management system, especially for densely populated areas, even though the sewers exist, many households choose not to connect to them [9] in many areas due to a multitude of factors.

The household connection rate to sewer is associated with a range of factors, including social, financial, policy, and technical considerations, alongside individual sanitation behaviours, with reported barriers to connection comprising cost, potential property damage, absence of government mandates, and dissatisfaction with current wastewater management facilities [9,12–16]. For example, in Latin America, people were unwilling to connect to the sewer due to the monthly tariff and high cost of connection, along with not being motivated by the government [9]. The coverage of sewer in cities of South Asia and sub-Saharan Africa was less than 25% due to issues including limited network extension and a lack of mechanisms to involve poorer households [16]. Another study in Zambia showed that a considerable number of households were hesitant to take sewer connections due to a history of sewerage obstructions and flooding [17]. In Dhaka, the capital of Bangladesh, only 20% of the total population is connected to a sewer, mostly from high-income communities [18]. The majority of households use some form of on-site storage to collect wastewater, and wastewater is typically released into the environment largely untreated [19].

Some sewerage authorities have attempted to develop interventions that target behaviour change to encourage connection where sewer lines exist. These include financial incentives and subsidies to drive sustainable investment into building a sewer and motivating households to connect [9,15,20,21]. Social programmes and other communication strategies were also considered to improve sanitation [9,12,20,22–24]. By contrast, water and sanitation authorities in certain areas implement a penalty if households fail to connect with the sewerage, or in some cases the government can take legal action if the faecal matter is discharged into the water sources [25]. The aim of this review is to understand the effectiveness of different behaviour change interventions across the globe in increasing the uptake of sewerage connections in households. The findings of this review can be used to implement context-specific, appropriate, and tailored interventions to be given as a comprehensive package in different regions of the world to increase the uptake of sewerage connection among households.

## Methods

### Overview

**Figure 1.**
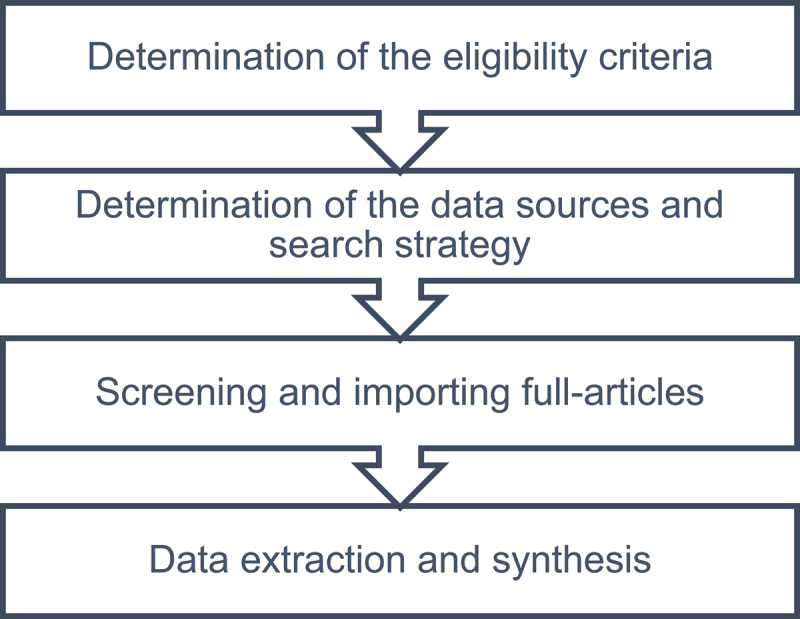
Flow diagram of the steps for conducting the scoping review.

The aim of this scoping review was to summarise the available evidence for the effectiveness of behaviour change interventions in promoting better household connectivity to sewers. We followed the PRISMA guidelines and incorporated the following steps during the review process (Figure 1) [26].

We developed a protocol that specified the research questions, inclusion/exclusion criteria, data sources and search engines (Supplementary Table S1). We followed the Preferred Reporting Items for Systematic Reviews and Meta-Analyses extension for Scoping Reviews (PRISMA-ScR) checklist to conduct the review [26].

## Types of publications, population, and interventions

We included journal articles and organisational or institutional professional or technical reports written in English in the review. In the latter category, we selected publications that focus on aspects of off-site sanitation involving sewer and behaviour change interventions provided to increase sewer connectivity (Supplementary Table S2). All eligible studies conducted across the globe covering both urban and rural populations were included.

Behaviour change intervention is defined as ‘a coordinated set of activities designed to change specified behaviour patterns’ [27]. All the included articles mentioned different interventions to connect the households to the sewer while implementing their projects. These interventions comprised subsidies, promotional activities, educational interventions (trainings/workshops/educational kits), and community engagement [9,20,28–31]. Therefore, these were considered as behaviour change interventions in this review.

We considered two types of subsidies: direct and indirect. Direct subsidies take the form of a monetary transfer to a specific beneficiary or household. The household is then able to spend that money on the goods or services of their choice. Indirect subsidies are those where the household or individual receives something which has monetary value but does not receive cash. The most common form of indirect subsidy of concern here is the reduction of the cost of necessary products or services such as costs of connecting to a network [32]. Subsidies may equal the full cost of the goods or services, in which case they are provided ‘free at the point of use’. A common example of this is that connection fees are waived for specific households. Therefore, we are referring to this indirect subsidy as a free connection that was received by the household owners while providing the sewer intervention.

## Outcome measures

The change in the number of households connected to the sewer after implementing behaviour change interventions was selected as the outcome measure.

## Inclusion and exclusion criteria

A set of specified inclusion criteria was determined that was used for searching studies relevant to the research objectives. The inclusion criteria involved selecting any journal article, organisational or institutional report and materials from websites that were related to behaviour change interventions for increasing sewer connection across the globe. The specified time frame used for searching relevant published studies was from 1 January,1980 to 31 December 2022, as the study protocol was developed in January 2023.

A set of exclusion criteria was developed and applied to the search (Supplementary Table S1). Articles that were not related to off-site sanitation and focused on on-site sanitation were excluded. Articles and reports published in any other language than English were not selected for further screening. Furthermore, any type of review article was excluded. We also excluded any article that did not contain full details of the programme design and outcome. The study eligibility criteria are summarised in Supplementary Table S1.

### Data sources and search strategies

Two reviewers (AR and SN), under the guidance of MUA developed a search strategy and independently searched PubMed, Cochrane, Scopus, and ProQuest through Hinari databases for peer-reviewed literature published from 1 January 1980 to 31 December 2022. For grey literature, Google, Google Scholar, and the websites of the World Bank, WSUP, Practical Action, and WaterAid were searched. The first 300 results were screened for grey literature search in Google and Google Scholar [33]. Manual searching of the reference list of the included articles was conducted for additional relevant publications.

We conducted a preliminary search for published scientific literature on the topic of interest to identify keywords for developing an advanced search. Afterwards, we developed key search terms related to households, sewers, behaviour change interventions, promotion, and effectiveness. A librarian working at the International Centre for Diarrheal Disease Research, Bangladesh (icddr,b) was invited to review the search strategy, and according to his suggestion, the search strategy was modified. A detailed search strategy with results is presented in Supplementary Table 3.

### Screening and importing full articles

According to the PRISMA guidelines, we selected articles in three phases: (i) identification, (ii) screening, and (iii) inclusion. The literature identified by the search terms was imported to EndNote (version 20), and duplicates were removed. The updated list was then imported into Rayyan’s online software.

MUA, AR, and SN collaboratively reviewed the articles by screening the titles and abstracts according to the study inclusion and exclusion criteria. The reviewers conducted a second review of each article that was excluded after the screening process to ensure that no pertinent papers had been inadvertently rejected. Studies that did not meet the criteria due to insufficient information in the title and abstract were referred to the reviewers for further discussion prior to a determination of inclusion. After that, shortlisted articles were screened in full text. Disagreements regarding eligibility were resolved through discussions among the two reviewers, with approval from the third reviewer. Corresponding authors were contacted in the situation when full-text articles were not found.

### Critical appraisal of the included studies

For our scoping review, we adapted the JBI Critical Appraisal Checklist for Case Reports to better fit our study [34]. The modified tool assessed eight key aspects: (1) population demographics, (2) history of sewerage conditions with a timeline, (3) current household connections, (4) clarity of methods and results, (5) description of interventions, (6) post-intervention conditions, (7) adverse events or limitations, and (8) key lessons. Two (AR, SN) reviewers assessed each case report against these criteria to ensure reliability and minimise bias. Any discrepancies between the reviewers were resolved through discussion and a consensus score was reached for each study. This critical appraisal allowed us to evaluate the overall quality and completeness of the included case reports, providing insights into the methodological strengths and limitations within the existing literature.

Each criterion was rated as ‘Yes’ (1 point), ‘No’ (0 points), ‘Unclear’ (.5 points), or ‘Not Applicable (N/A)’, with N/A responses excluded from the final score calculation. In the context of each study, a score of 1 or ‘yes’ considered as good quality, a score of 0.5 or ‘unclear’ considered as fair quality and a score of 0 or ‘no’ considered as poor quality [35]. The NHLBI defines a study as ‘good’ when it exhibits low bias, resulting in enhancing the likelihood that its results are accurate and genuine. A ‘fair’ study recognises certain biases; yet, these biases are insufficient to invalidate its conclusions. A ‘poor’ rating suggests a significant risk of bias, consequently questioning the accuracy of the results [36]. The overall critical appraisal for each study was determined by summing the individual scores assigned to the eight criteria. Case report quality was classified as high quality (≥6), moderate quality (4–5), and low quality (<4) based on the total score.

### Data extraction and synthesis

A data extraction form was created. The data were extracted based on the initial author’s last name, the article category, the guiding criteria, and the number of checklist items. The review checklist was extracted into an Excel spreadsheet. Title, objectives, methodology, results, references, and recommendations were the section categories that were used for the review checklist.

This data matrix was disaggregated into two key themes with relevant sub-themes, including (i) interventions provided to increase sewerage connection among households and (ii) the impact of the interventions for sewerage connection among households. Two reviewers extracted data on each theme under the guidance of M.U.A. Subsequently, a narrative synthesis of the extracted data was performed.

### Ethical approval

The study involved summarising existing published data from the literature. No ethical issues arose from the execution of this work.

## Results

The initial literature search yielded 10,017 unique articles, of which 109 were duplicates. These were assessed to determine against the inclusion criteria, resulting in a set of 33 relevant articles. Of these 33 articles, only 11 met the eligibility criteria and were included in the review (2). Reason of the exclusion of the articles was mentioned in the supplementary Table 3.

**Figure 2.**
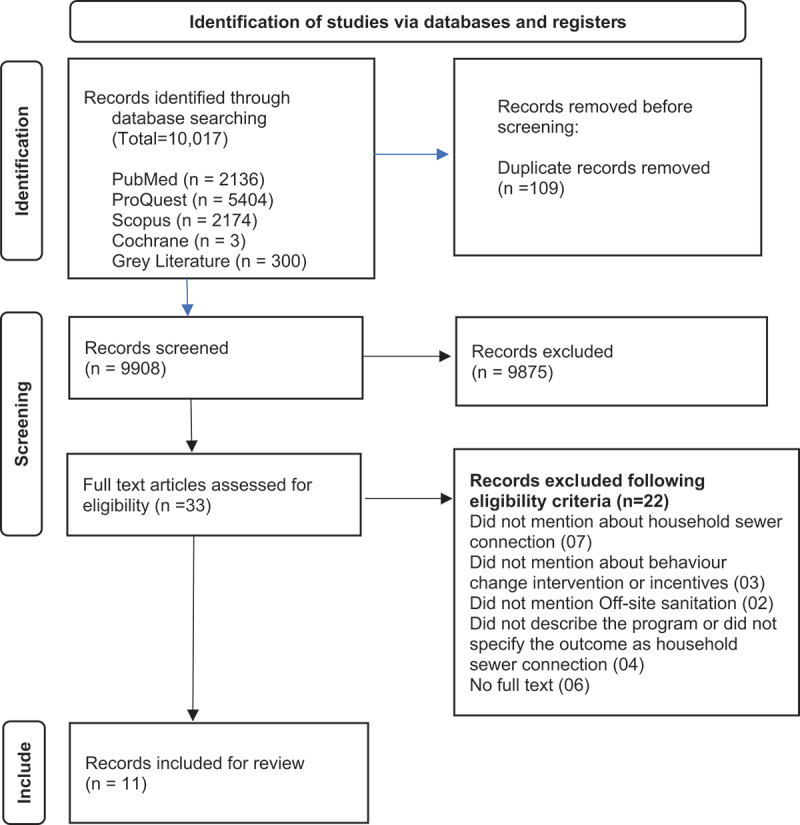
PRISMA flowchart illustrating the study selection process.

The studies were conducted in diverse locations, both upper- and low-income countries, including Ecuador, Colombia, Bolivia, multiple cities in Brazil, Kenya, Morocco, India, and Pakistan. One report from the World Bank [9] included several case studies from multiple locations. Among those case studies, five met the inclusion criteria of this review [9] and were included as five distinct studies. The selection process is summarised in Figure 2. The studies are summarised in Table 2.

**Figure 3.**
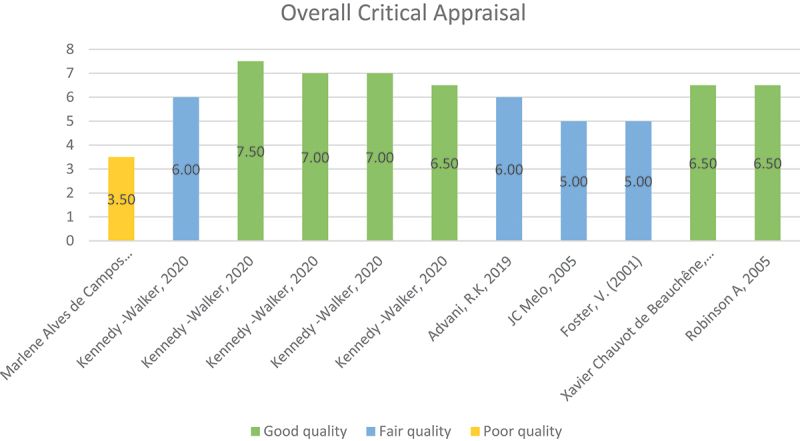
Distribution of overall study quality appraisal.

The quality assessment of 11 studies were classified as good, fair, and poor quality according to their total critical appraisal ratings. Of these, six studies were assessed as high quality, with ratings between 6.50 and 7.50. Four studies were classified as fair quality, with scores ranging from 5.00 to 6.00. One study was deemed of poor quality, obtaining a score of 3.50 (Figure 3). The distribution reveals that the majority of research were categorised within the good to fair quality range, but only one study exhibited poor methodological rigor. The full assessment can be found in Supplementary Table S4.

### Design and settings of included projects

The majority (9 out of 11) of the studies were in professional or technical project reports, with all being overviews of implemented projects. Among the included studies, six (55%) were conducted in upper-middle-income countries [9] of the region of Latin America, and the remaining five studies (45%) were conducted in low-income countries of different regions of the world. Ten (90%) of the included studies were implemented in urban settings, and only one (10%) was implemented in rural settings.

### Summaries of included projects

The case study from Lodhran, Pakistan, the local NGO Lodhran Pilot Project (LPP) implemented a low-cost sewerage schemes project in Punjab in 2001 to connect rural households to the sewer across 12 villages which had equal or less than 1200 households [29]. The project focused on connecting households to sewers in these villages that had no existing sewer connection. In India, Tamil Nadu was the most urbanised state of the country, where 75% of households had access to on-site sanitation, and only one-fifth of the population of the state’s capital had a connection to the sewer [9]. Thus, A project called Third Tamil Nadu Urban Development Project (TNUDP III) targeted to connect 1,551,995 households to sewers in 25 cities statewide between 2005 and 2014.

Among the studies in Brazil, one followed a mixed-method design to review the implementation barriers of the ‘Connect to the Network’ programme in the state of Parana. ‘Connect to the Network’ encompasses 17 projects and primarily focuses on establishing guidelines for social and environmental interventions to increase the uptake of sewer connections [30]. Another programme called ‘Se liga na rede’ in the western and southern parts of the Sao Paulo metropolitan region was designed to connect around 192,000 households to the new sewer between 2012 and 2018. In 2012, this programme, which the state enterprise started, was designed to accelerate the expansion of sewer connections in the Greater Vitória Metropolitan Region (GVMR) of the state of Espirito Santo. This pilot programme ran for three years (2012–2015) and aimed to connect 20,000 households to the sewer. One of the aspects of the programme was that they specifically targeted low-income households to increase sewer connections from the existing 13,000 connections [9].

A condominial sewerage approach was initiated under the ‘Bahia Azul’ (Blue Bahia) umbrella programme in the state’s capital city of Salvador and 11 other cities between 1995 and 2004. The programme aimed to enhance solid waste, water, and sewerage solutions for all urban residents, with special attention paid to those residing in low-income informal settlements where traditional sanitation methods could not be used. In Salvador, only 26% of the population had access to sewer prior to the programme. At the beginning of the programme, the condominial sewerage approach was only used for low-income areas; however, with its success, the model was adopted for all areas of the city [20].

A similar approach was initiated in the urban cities of La Paz and El Alto, Bolivia. Learning from Brazil, the El Alto Pilot Project (EAPP) aimed at implementing the condominial sewerage and tested its applicability in the context of private sector participation in service provision. The short-term pilot project’s objective was to provide water and sanitation connections to 5,000 poor households, where 60% of the households lived below the poverty line. The pilot project was implemented from 1998 to 2000 [28].

In the urban area of Guayaquil, Ecuador, a simplified sewerage pilot project enabled connections in hard-to-reach areas and got beyond the technical difficulty of joining households to a sewer. The target audience for the programme resided in the most impoverished areas, where the number of residents living in poverty ranged from 55% to 70%, and 18% to 32% of households experienced extreme poverty. The duration of the pilot project was two years (2013–2015) [9].

The National Administrative Department of Statistics (DANE) of Columbia estimated that 93% of Colombia’s urban population has access to sewer, with 78% of those in the poorest quintile having sewer access. In 2011, the government approved the National Development Plan, which sauthorised subsidies for household connections. This initiative led to the ‘Connect-with-water program’, aimed to connect 90,000 poor families by providing subsidies. The programme installed sewer connections in households across 20 municipalities from 2012 to 2014 [9].

The Global Partnership on Output-Based Aid (GPOBA), a World Bank–administered programme, piloted an innovative Output-Based Aid (OBA) method with the goal of increasing access to water and sanitation services among the underprivileged living in urban and peri-urban areas of Morocco. The project launched in 2007 and aimed to connect 11,300 households (approximately 56,000 people) to piped water and sanitation services in poor peri-urban neighbourhoods in the three cities (Casablanca, Tanglers, Meknes) [31].

Another Output-Based Aid (OBA) Program (2012–18) was launched in the Nairobi City of Kenya, to facilitate sewer connections among low-income households living in informal settings. New household sewer connections were constructed under the project, and the programme targeted around 167,000 people or 13,000 households to connect to the sewer to provide better sanitation [37].

### Interventions provided to increase sewer connections among households

The included projects used different behaviour change interventions that influenced the uptake of sewer connections among households in different communities (Table 1). The interventions involved indirect subsidies (free connection) to increase connectivity, promotional activities (door-to-door campaigns, awareness campaigns, promotion of programme benefits), education (training or workshop, or educational kit distribution), and community engagement activities (mass mobilisation for construction and maintenance).

**Table 1.** Interventions provided for increasing sewer connection among households.

Reference	Country	Interventions					
		Indirect subsidies(Free connection)	Promotional activities	Education			Community engagement
				Training	Educational kit	Workshop	
Marlene Alves de Campos Sachet, (2020) [30]	Brazil (Parana)			✓	✓		✓
Kennedy -Walker, 2020 [9]	Brazil (Espirito Santo),	✓	✓	✓	✓		✓
Kennedy -Walker, 2020 [9]	Colombia	✓					
Kennedy -Walker, 2020 [9]	Ecuador	✓	✓	✓			✓
Kennedy -Walker, 2020 [9]	Brazil (Sao Paulo)	✓	✓				
Kennedy -Walker, 2020 [9]	India (Tamil Nadu)		✓				
Advani, R.K, 2019 [37]	Kenya	✓	✓				✓
JC Melo, 2005 [20]	Brazil (Salvador)	✓	✓	✓		✓	✓
Foster, V. (2001) [28]	Bolivia	✓			✓		✓
Xavier Chauvot de Beauchêne, World Bank, 2011 [31]	Morocco	✓	✓				
Rural, S.U., 2005. Sanitation in South Asia [38]	Pakistan						✓

## Free connection from households to sewer network

Seven projects (Table 2) mentioned providing a free connection from households to the sewer and maintenance fees with other interventions to increase the number of households connecting to the sewer [9,20,28–31,37]. In Espirito Santo and Morocco, households were connected to the sewer free of charge [9,31], whereas in Salvador, with free connection, technical assistance was given [20].

**Table 2.** Summary of behaviour change interventions provided to households for connecting to sewer networks.

Data source (Author, Year)	Country	Intervention						Data source type of the study	Size of the target population	Total cost	Outcome (Rates of sewer connection increased)	Reasons behind connecting to the Sewer network	Recommendations
		Free connection	Promotional activities	Education			Community engagement						
				Training	Educational kit	Workshop							
Marlene Alves de Campos Sachet, (2020) [30]	Brazil (Parana)	_	_	Training for plumbers and stonemasons residing in the area of the project to work with the community in servicing home connections	Didactic materials were distributed regarding important issues on environmental sanitation (water, sewerage, garbage, health)		Social technical work was carried out among the citizens in order to describe the ‘Connect to the work’ programme benefit and their role	Primary	13,286 households from 17 projects	_	The overall average of regularised connections was 78.4%	_	Socio-environmental initiatives carried out within the framework of the “Connect to the Network” program be compared with similar initiatives that are planned and executed with sanitation companies
Kennedy -Walker, 2020 [9]	Brazil (Espirito Santo),	Build free connection of internal plumbing from homes to inspection chambers	Door-to-door campaigns to persuade the population to connect to the network	Training of more than 130 private installers	Community education activities		Mass mobilisation	Primary	In Brazil (Espirito Santo), 20000 households	In Espírito santo (es), Brazil, us$8.7 million	The number of household sewerage connections rose from about 13,000 to about 33,000 during that time.	Training operation and maintenance teams to use appropriate techniques for services within these urban blocks. The training covered social aspects and learning to relate to residents	Application of municipal regulations and laws can work even in the absence of subsidies and other economic and financial incentives
Kennedy -Walker, 2020 [9]	Colombia	Government entities received permission to cover the full cost of household access to sewerage connections.							The initial goal of the program was to connect 90,000 families with water and/or sewerage.	In Colombia, the first phase—us$41 million.	In Colombia, sewerage connections were installed in 30,159 homes across 20 municipalities in its first phase of implementation. More than 30,000 connections resulting in 75% connection to sewers		Working in individual homes has technical and social complications. Each home and each client is different and requires individualised responses.
Kennedy -Walker, 2020 [9]	Ecuador	The household connection was free of cost. The total cost of connection was estimated at US$500 per household	Promotion of programme benefits	Promoted training courses for community leaders to strengthen local capacity for self-management.			Contractors and inhabitants signed agreements outlining the scope of work, allowing inhabitants to authorise the proposed project, assess the quality of delivered outputs, and accept or reject the final product in writing.			Guayaquil, Ecuador, us$146.5 million	In Ecuador, approximately 40,000 new connections were to be completed. Improving the city’s connection rate from 45% to 85%. In Ecuador, 10,000 households Benefitting from the sewerage connection		
Kennedy -Walker, 2020 [9]	Brazil (Sao Paulo)	Free Connections are key to encouraging low-income households to connect	Carries out social promotion activities in identified neighbourhoods					Primary	Connecting 192,000 households	São Paulo, Brazil, US$115 million	From 2012 to 2020, the program connected 35,637 properties among 192,000 properties, which is 19% of the targeted population.	Since 2002, it has been mandatory in the city of São Paulo to connect to a sewerage network if it is available.	Connection and tariff subsidies are key to encouraging low-income households to connect, particularly if repair costs of damaged floors are included.
Kennedy -Walker, 2020 [9]	India (Tamil Nadu)		Provide efforts to educate people on the need for awareness campaigns; meetings at the ward level with active involvement of elected representatives, ULB staff, and other influencers; issuing pamphlets; engaging nongovernment organisations for facilitation.					Primary	Connecting 1,551,995 households statewide	639,104 households completed internal plumbing and connected to the sewerage network	Sewerage projects have become a reality in 35 cities, work is progressing in 20 cities, and projects are in the preparation stage in five cities	Safely manage human excreta and wastewater through a sewerage network and sewage treatment in phases in cities and towns. Regulatory provisions in Tamil Nadu (TN) mandate that a house service connection be made when a sewerage line is laid and available within 100 meters from a household. » This provides the legal framework for urban local bodies to push households to connect to sewers	Even after the public deposit contribution, the city is not able to meet its project cost. So, contributions from the ULB or additional grant money from the state government are needed to fulfil the project.
Advani, R.K, 2019 [37]	Kenya	Provided free connection to low-income communities to connect their households to the sewerage	Customer awareness building _	_	_	_	Focus group discussions with potential customers, which were carried out before the contractor entered the areas of intervention, helped to increase community ownership	Primary	137,243 people	In Nairobi, Kenya US$10.55 million	The project reached 137,243 people through the provision of 9,843 household sewerage connections.		The success of the connection program depended on achieving a balance between expanding services through reducing upfront connection costs.
JC Melo, 2005 [20]	Brazil (Salvador)	Households received free connection	Environmental education	_		Creation of a utility-embedded social mobilisation unit and extensive community mobilisation efforts	Involving public organisations, schools and communities to get the wider public and inhabitants of the city participating and committed to the goals of the program	Primary	2.5 million people	In Salvador Metropolitan Region, Bahia state, northeast Brazil, US $450 million went toward sewerage	The program officially ended in 2004; coverage had increased to 60%	The state government created Law 7307 of 1998 to mandate households to connect to sewerage	
Foster, V. (2001) [28]	Bolivia	Households received free connection			The purpose of the hygiene education component was to provide moral and technical support for households to adopt modern hygiene practices, in particular by helping them to construct their own bathrooms and associated facilities such as kitchens and laundry sinks. Without such investments within the home, a sewerage connection brings little or no benefit to households and has been shown to have virtually no impact on water consumption.		Provided training to the community people for constructing and maintaining the condominial network. Community involvement helps to improve the acceptability of the infrastructure, promotes network connections and provides an entry point for imparting hygiene education.				4,050 households in nine neighbourhoods of El Alto connected to condominial sewerage after the completion of the pilot project		
Xavier Chauvot de Beauchêne, World Bank, 2011 [31]	Morocco	Households located in selected areas were eligible for free connection	Operators also developed new means of reaching potential customers by sending dedicated teams to marketplaces or to the heart of targeted neighborhoods to record demand from beneficiaries who might not easily travel to one of the operator’s agencies						Supply of water to a total of 10,504 households and sanitation services to a total of 9,036 households, benefitting more than 52,500 people		The pilots experienced a slow start, with about 2,000 connections (15% of the program’s three-year objective) in the first year, but connection rates doubled in the second year	Direct benefits to households in terms of time savings, reduced health costs and improved hygiene practices	
Rural, S.U., 2005. Sanitation in South Asia [29]	Pakistan						The responsibility for providing the components is shared: construction of the internal components is financed and managed entirely by the community; provision of the external components, technical assistance, hygiene promotion, and social guidance are the responsibility of LPP.		In 12 villages Connected 1200 households				

In Colombia, government entities have received permission to cover the total cost of household access to sewers. This initiative influenced all types of households to connect to the sewer. Consumers received an upgrade of internal and external sanitation facilities, with an investment of US$2500 per targeted household from the service provider. This intervention connected 75% of the targeted households without sewer connection (30,159 among 40,000 households) across 20 municipalities of the country [9].

## Free connection and promotional activities

Two studies conducted in Sao Paulo, Brazil, and Morocco provided a free connection from households to sewers and promotional activities to motivate people to connect their houses to the sewers [9,31]. All of these countries have unique and different socio-economic and geographical contexts.

In Sao Paulo, free connection to the household, along with promotional activities, was provided. This free connection service includes the installation of internal connections to transport graywater and wastewater to the grid, laying pipes and fittings, building inspection chambers, establishing the connection, and replacing damaged floors for low-income families. However, though the programme offered both free connections and conducted promotional activities, it could only connect 19% of the targeted population (35,637 properties out of 192,000 properties). The reported primary reason behind the low uptake of sewer among households was the existing water crisis [9].

In Morocco, the sewer connection programme, with a combination of both free connection service to the houses and promotional activities, reached 9,036 households (80% of the targeted population) for sanitation services in several cities among 11,300 (targeted) households, benefitted 52,000 people [31].

## Free connection, promotional activities, and community engagement

A study conducted in Kenya provided free connections to low-income communities, promotional activities for customer awareness, and community engagement to increase community ownership. Due to this amalgamation of interventions, the programme reached 9,843 households among the targeted 13,000 households (76%), providing 37,243 people among 167,000 people (82% of the targeted population) with connection to the sewer [37].

## Free connection, promotional activities, community engagement, and educational interventions

Three programmes [9,20] provided a combination of free connection to the houses, promotional activities, educational interventions (training or workshop, or educational kit distribution), and community engagement for the uptake of sewer connection. In Espirito, Brazil, free connection, door-to-door campaigns, training for private installers, and mass mobilisation were provided to low-income communities. The programme built a free connection of internal plumbing from homes to inspection chambers. This resulted in an increase in the uptake of sewerage connections from 13,000 households to 33,000 households (54%) [9].

In Ecuador, the project provided free connections to low-income communities, promoted programme benefits, trained community leaders, and ensured community participation in the targeted area. The estimated total cost of the connection was US$500 per household. This amount included cosigning and sanitising the existing sanitation solution, building the inspection chambers, directing all the drains of the property to the sewer, and physically connecting to the network. After the interventions, the city’s sewer coverage improved by 40%, and its household connection rate reached 85%. This benefitted approximately 10,000 households in economically disadvantaged districts [9]. In Salvador, Brazil, just 26% of the population had access to sewers in 1995. The project engaged public organisations, schools, and community people in the environmental education programme to motivate inhabitants to connect their households to the sewer. Also, the sewer connection was made free for the residents. As a result, the coverage reached 60% by the time the programme officially ended in 2004, with a 34% increase in household connection rate [20].

## Behaviour interventions: community engagement and education

In two studies, educational interventions (training, workshops, or distribution of instructional kits) were combined with community engagement to motivate the people to connect their households to the sewer. One is the Connect to the Network programme in Parana, Brazil, which is focused on community engagement activities to promote the benefits of the programme. They also provided training to the plumbers in the project area regarding water, sewer, garbage, and health. As a result, 10 out of 17 projects under this programme succeeded in achieving 80% of household connections to the sewerage network [30]. Similarly, in Bolivia, hygiene education was provided to adopt modern hygiene practices, and training was given to the local people to construct and maintain the condominial sewer. Following that, 4,050 households (81% of the targeted households) in nine neighbourhoods of El Alto were connected to condominial sewerage after the completion of the pilot project [28].

## Behaviour interventions: community engagement

The Lodhran Pilot Project (LPP) of Pakistan engaged the community by involving them during the development and maintenance of the sewer and also provided social guidance. The average per-household cost was about US$72. LPP bore the external cost (construction of the main sewer and disposal station), and the local people had to bear the internal cost (household connection and chamber), which was about 50%. By using this community engagement approach, LPP managed to motivate people to connect to the sewer network, and about 1200 rural households in the study area were connected annually, where the existing sanitation situation was critical [29].

## Behaviour interventions: promotion

In India, promotional activities involving local elected representatives of communities and influencers were implemented to motivate households to connect to the sewer. These helped the connection of 40% of the households (639,104 among 2,613,189 properties) to the sewer in 35 cities [9].

## Impact of different interventions

**Figure 4.**
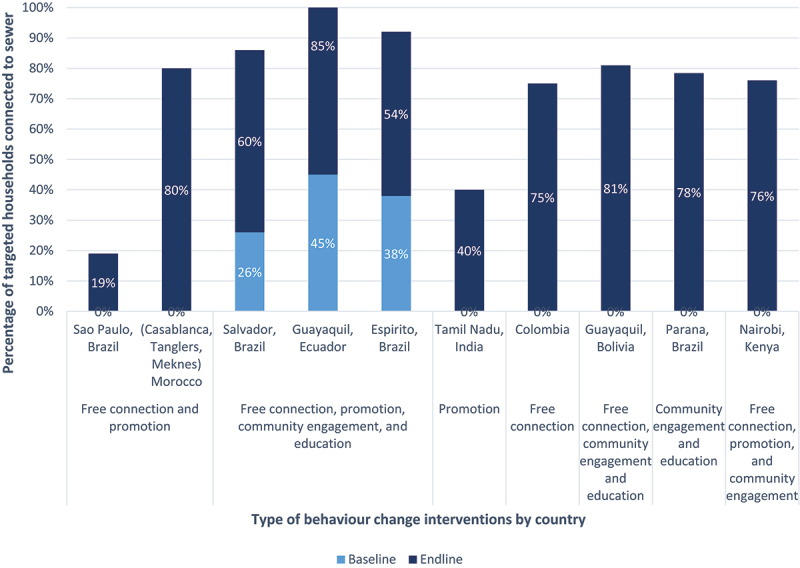
Percentage of households connected to sewer network at the end of the intervention.

Figure 4 illustrates the effectiveness of different behaviour change interventions in improving sewer connections from baseline to the end line in targeted households across different countries. The results demonstrate that the intervention package encouraged people to connect their households to the sewer by offering free connections, promotional efforts, and involving the community. This effectiveness is evident from findings in Colombia (75%) [9], Morocco (80%) [31], and Kenya (76%) [37], where sewer connections significantly increased following interventions. A sewer project conducted in Colombia successfully attained a 75% rate of connectivity to the sewer within its intended population. This achievement was facilitated via the implementation of sewer infrastructure and complimentary household connections to the sewer, which were made available to all types of households [9]. Additionally, Morocco is categorised as a lower-middle-income country (LMIC) that has expanded the number of homes linked to the sewer by implementing a strategy that includes providing free connections to houses and implementing promotional campaigns aimed at households of various income levels. The high success rate of this project in reaching 80% of the intended population can be due to its extensive targeting of all sorts of households and provision of free sewer connection from households to the sewer [31].

The incorporation of free connectivity, along with the execution of targeted promotional activities and community engagement initiatives, showed strong results in terms of connecting households to the sewer. This is especially apparent in low- and middle-income countries (LMICs) such as Kenya. Kenya implemented an intervention package that included providing free sewer connections to specific households, conducting promotional efforts, and community engagement activities. This package notably focused on low-income families and effectively connected the highest proportion of households to the sewer [37]. Likewise, the incorporation of educational resources (such as training, educational kits, and workshops) along with free access to sewers, promotional initiatives, and community involvement led to a substantial enhancement in the provision of sewer connections. Salvador, Brazil; Espirito, Brazil; and Guayaquil, Ecuador, experienced significant enhancements in terms of connecting targeted houses to the sewer through the implementation of multimodal intervention strategies [9,20].

## Discussion

This review aimed to identify the effectiveness of different behaviour change interventions to promote household connectivity to sewers. The synthesised evidence from this scoping review indicates that providing interventions involving free connections to households along with promotional activities yielded significant outcomes. Additionally, in the context of LMICs, combining community engagement with indirect subsidies encouraged people to connect their households to the sewer, indicating the need for a combined behaviour change intervention package for achieving a greater percentage of sewer uptake.

Eight of our cases [9,20,28,31,37] provided a free connection with or without implementing promotional and community engagement activities to increase the uptake of sewer connections among households. Providing free connections that cover the costs associated with the construction of infrastructure, connection, and maintenance fees influenced the community to a greater extent in connecting their households to the sewer. From our review, in Colombia, providing only free connections significantly improved the uptake of sewer connections [9]. Similarly, most of the other projects that have incorporated free connection in the intervention showed the most significant rise in sewer uptake. Even in Bangladesh, people from low-income communities were willing to connect their toilets to the sewer only if five conditions were met, mostly emphasising the no installation cost for sewers [18]. This emphasises the significance of providing financial support to households for connection and maintenance when developing sanitation solutions for extremely impoverished populations, which largely impact the uptake of sewer connection.

Along with free connection, providing interventions involving community participation has a larger impact on low-income communities. In Bolivia, engaging the community with a free connection to the households resulted in 80% of the intended households being connected to the sewer. The higher connection rate resulted from the community’s training in constructing and maintaining the condominial sewer, which improved the acceptability of the infrastructure and created ownership among them. The households did not bear the cost of infrastructure and provided labour in lieu of money, which improved the connection rate to the sewer [28]. Similarly, a study conducted in Ghana explored that sanitation vouchers for toilet construction to stop open defecation were effective. Voucher-eligible households received a voucher covering the total costs of a durable latrine substructure, which included a durable slab and pit lining. Households were responsible for digging the pit and building the superstructure (themselves or with help). This accessible latrine construction voucher and community engagement decreased open defecation, particularly among those who received the interventions [39]. Other studies also showed that factors including sewerage fees, financial affordability, and involvement of local residents improve sewerage connection uptake and may ensure the intervention’s long-term sustainability [40–43]. This indicates that the cost of sanitation and involving the beneficiaries has a more significant outcome and motivates people since it builds ownership among them, starting a chain reaction of more intervention uptake.

While promotional activities can positively influence the uptake of sewer connections, relying solely on promotion without the inclusion of additional behaviour change intervention may yield limited results. In Pakistan and Tamil Nadu, India, people needed to bear the cost of establishing a connection between their households and the sewer and subsequent expenditures of upkeep [29]. Although the organisation provided funding for sewer construction, the exorbitant expenses associated with connecting materials and maintenance posed a significant barrier for low-income residents. As a result, a considerable portion of this population had financial constraints, resulting in a decrease in the number of people in this group who could connect to the sewer [29]. This indicates that only promotion without some level of monetary benefits might not be fruitful. This emphasises the significance of considering not only the initial expenses of building but also the financial responsibility of households for connection and upkeep when developing sanitation solutions for low-income communities.

In conclusion, the evidence across various countries highlights the critical role of both financial and community engagement strategies in increasing the uptake of sewer connections among low-income households. Programmes that provided free connections alongside community engagement activities showed significantly higher connection rates. This indicates that eliminating financial barriers and fostering a sense of ownership through community involvement can enhance the acceptability and sustainability of sanitation infrastructure. Conversely, initiatives that lacked comprehensive financial support struggled to achieve similar success. Therefore, to effectively address sanitation challenges in low-income communities, it is imperative to design comprehensive programmes that integrate free connections or financial subsidies, as well as robust community engagement. This holistic approach ensures that the infrastructure is not only built but also utilised and maintained, leading to sustainable improvements in public health and hygiene.

## Limitations

Inferences drawn from our review are limited to only eleven cases, with no RCT, and no study has checked the effectiveness of any particular intervention on the uptake of household sewer connectivity. These facts limit the quality of evidence; however, they highlight how this topic is understudied, given its potential policy importance. Therefore, we could not generate granular evidence on the precise BCTs (Behaviour Change Techniques) and their relative effectiveness in influencing households to connect to the sewer network. Instead, we focused on the comprehensive description and synthesis of overall interventions to increase the uptake of household connection to sewer and their impact in a generalised context.

## Recommendation

The analysis of our scoping review reveals that applying an intervention that incorporates the two components, free connection or financial aid, with community-engagement activities has better potential to motivate households to connect to the sewer. The evidence also indicates that the effectiveness of the interventions may vary depending on the households’ geographical location, cultural context, and socio-economic conditions, which must be dissected for policymakers and intervention delivery partners. Following that, we propose to develop a comprehensive package of behavioural interventions incorporating financial aid and active community participation to have a larger uptake of sewer connections. Additionally, we recommend that prior to developing methods to promote connections to sewers, it is essential to thoroughly comprehend the economic context, including cost issues, financing sources, and appropriate usage of public funds. This method will ensure that initiatives are well-informed and strategically formulated, considering the distinctive socio-economic aspects of each community. This will also help to develop the appropriate community engagement methods, incorporating different activities, which would build ownership and ensure the sustainability of the connection. More rigorous approaches and studies, such as controlled trials or theory-driven evaluations, should also be used to help generate more reliable evidence. Therefore, more high-quality research is required to draw a more evidence-based rigorous inference about the context and resource-specific behaviour change interventions that can influence households to connect to the sewer.

## Supplementary Material

PRISMA ScR Checklist.docx

## Highlights

- Reviewed the effectiveness of different behaviour change interventions for sewer uptake
- Combining financial support with community engagement significantly improves the sewer connection rates
- Effectiveness varies based on geographical location, cultural context, and socio-economic conditions
- More high-quality research is needed to identify the most effective behaviour change techniques across diverse settings
